# A novel mutant of the Sup35 protein of *Saccharomyces cerevisiae *defective in translation termination and in GTPase activity still supports cell viability

**DOI:** 10.1186/1471-2199-9-22

**Published:** 2008-02-11

**Authors:** Céline Fabret, Bruno Cosnier, Sergey Lekomtsev, Sylvie Gillet, Isabelle Hatin, Pierre Le Maréchal, Jean Pierre Rousset

**Affiliations:** 1 IGM, Univ Paris-Sud, UMR 8621, Orsay, F 91405, France; 2 CNRS, Orsay, F 91405, France; 3 Engelhardt Institute of Molecular Biology, The Russian Academy of Sciences, 119991 Moscow, Russia; 4 IBBMC, Univ Paris-Sud, UMR 8619, Orsay, F 91405, France

## Abstract

**Background:**

When a stop codon is located in the ribosomal A-site, the termination complex promotes release of the polypeptide and dissociation of the 80S ribosome. In eukaryotes two proteins eRF1 and eRF3 play a crucial function in the termination process. The essential GTPase Sup35p, the eRF3 release factor of *Saccharomyces cerevisiae *is highly conserved. In particular, we observed that all eRF3 homologs share a potential phosphorylation site at threonine 341, suggesting a functional role for this residue. The goal of this study was to determine whether this residue is actually phosphorylated in yeast and if it is involved in the termination activity of the protein.

**Results:**

We detected no phosphorylation of the Sup35 protein *in vivo*. However, we show that it is phosphorylated by the cAMP-dependent protein kinase A on T341 *in vitro*. T341 was mutated to either alanine or to aspartic acid to assess the role of this residue in the activity of the protein. Both mutant proteins showed a large decrease of GTPase activity and a reduced interaction with eRF1/Sup45p. This was correlated with an increase of translational readthrough in cells carrying the mutant alleles. We also show that this residue is involved in functional interaction between the N- and C-domains of the protein.

**Conclusion:**

Our results point to a new critical residue involved in the translation termination activity of Sup35 and in functional interaction between the N- and C-domains of the protein. They also raise interesting questions about the relation between GTPase activity of Sup35 and its essential function in yeast.

## Background

The translation of genetic information into proteins is essential for all biological systems. In eukaryotes, the process is divided into at least three steps: initiation, elongation and termination, and all three steps of translation involve GTP-binding proteins and phosphorylations [1,2]. The structure of the GTP-binding proteins functioning at each step is well conserved from yeast to mammals, and these proteins are fundamental to living cells [3]. In the initiation and elongation steps, eIF2 and eEF1A, which deliver, respectively, the methionyl-initiator tRNA to the 40S ribosomal subunit and the aminoacyl-tRNAs to the A-site of the ribosome, were identified as the GTP-binding proteins [4] while in the termination step, it is eRF3 [5,6].

Translation termination takes place on ribosomes when a stop codon enters the ribosomal A site and signals polypeptide chain release from the peptidyl-tRNA located in the ribosomal P site. In eukaryotes, two polypeptide chain release factors have been described: eRF1 recognizes and decodes all three nonsense codons and eRF3 stimulates peptidyl-tRNA hydrolysis in the ribosome in a GTP- and eRF1-dependent manner [7-9]. Recent genetic and biochemical data suggest that the GTPase activity is required to couple the recognition of translation termination signals by eRF1 to efficient polypeptide chain release [9,10]. Furthermore, reconstitution *in vitro *of the eukaryotic translation initiation, elongation, and termination processes made it possible to propose a model for the mechanism of translation termination in eukaryotes. Binding of eRF1, eRF3, and GTP to pretermination complexes induces a major structural rearrangement that leads to GTP hydrolysis for correct positioning of eRF1, followed by rapid release of the nascent peptide [9]. Similarly, in prokaryotes, RF3 is involved in recycling of RF1 and RF2 [11].

In the yeast *Saccharomyces cerevisiae*, eRF1 and eRF3 are encoded by essential genes, *SUP45 *and *SUP35*, and often designated as Sup45p and Sup35p, respectively. eRF1 and eRF3 can interact both *in vivo *and in *vitro *[8,12-14]. The eRF3 genes are conserved from yeast to mammals. In most species examined, eRF3 consists of three domains (N, M and C) whose functions have been defined for *S. cerevisiae *eRF3. Both the N and M domains are dispensable for viability and translation termination [15] in contrast to the C-terminal region which carries the GTPase activity, interacts with eRF1 and is indispensable [16]. All the mutants isolated up to now, showed a correlation between GTPase activity and viability.

The C-terminal domain of the eRF3 proteins is highly conserved between species and shows significant homology [16], as well as close structural similarities [17] to the elongation factor eEF1A. In *S. cerevisiae*, and also in other budding yeast species, the N and M domains are responsible for the formation of the prion-like [PSI+] factor [18-21]. In [PSI+] (but not in [psi-]) cells, eRF3 forms aggregates which lead to defects in translation termination and consequently to omnipotent nonsense suppression. The N domains of higher eukaryotes have no obvious sequence homology with the corresponding domains of lower eukaryotes although, they have an unusual amino acid composition as do the yeast proteins [22].

In this study, we identified a potential cAMP-dependent protein kinase A (PKA) phosphorylation site in the C-terminal region of the yeast Sup35 protein. We show that the protein is phosphorylated *in vitro *by PKA on threonine 341 (T341), although no phosphorylation was detected in the conditions tested *in vivo*. Alteration of this evolutionary conserved site modifies the *in vivo *and *in vitro *activity of the protein in translation termination. Surprisingly our results demonstrate that Sup35p mutants displaying very low GTPase activity are able to support cell growth.

## Results

### Conservation of the phosphorylation site among organisms

**Figure 1:**
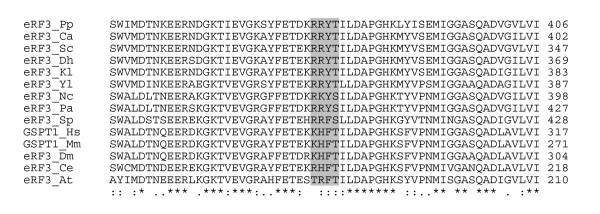
**Alignment of eRF3 protein sequences**. The 60 amino acids surrounding the T341 residue are shown, with the PKA phosphorylation consensus site shaded. Nc, *Neurospora crassa*; Pa, *Podospora anserina*; Sp, *Schizosaccharomyces pombe*; Sc, *Saccharomyces cerevisiae*; Kl, *Kluyveromyces lactis*; Dh, *Debarromyces hansenii*; Ca, *Candida albicans*; Pp, *Pichia pinus*; Yl, *Yarrowia lipolitica*; Hs, *Homo sapiens*; Mm, *Mus musculus*; Dm, *Drosophila melanogaster*; Ce, *Caenorhabditis elegans*; At, *Arabidopsis thaliana*.

The amino acid sequence R/K-R/K-N-S/T is the consensus PKA recognition site, and either S or T is the phosphorylation site. A putative PKA phosphorylation site containing threonine 341 (T341) is present in the *S. cerevisiae *eRF3 (Sup35p) sequence and is conserved among others yeasts and fungi. In drosophila, human and mouse the site is degenerated, but T341 still belongs to a phosphorylation site, recognized by the CK2 protein kinase (Fig. 1). In a sequence-based approach, the evolutionary conservation of all PKA consensus sites in the *S. cerevisiae *proteome was systematically assessed within a group of related yeasts [23]. The study demonstrated that the likelihood of phosphorylation correlated well with the degree of conservation of the PKA consensus sites. The evolutionary preservation of the eRF3 phosphorylation site thus suggests that this site probably plays an important role *in vivo*. To test the functionality of the PKA phosphorylation site of Sup35p, we generated threonine to alanine (A) or aspartate (D) mutants. Alanine cannot be phosphorylated, whereas aspartate mimics the presence of phosphate groups.

### Sup35p is phosphorylated *in vitro *by PKA

**Figure 2:**
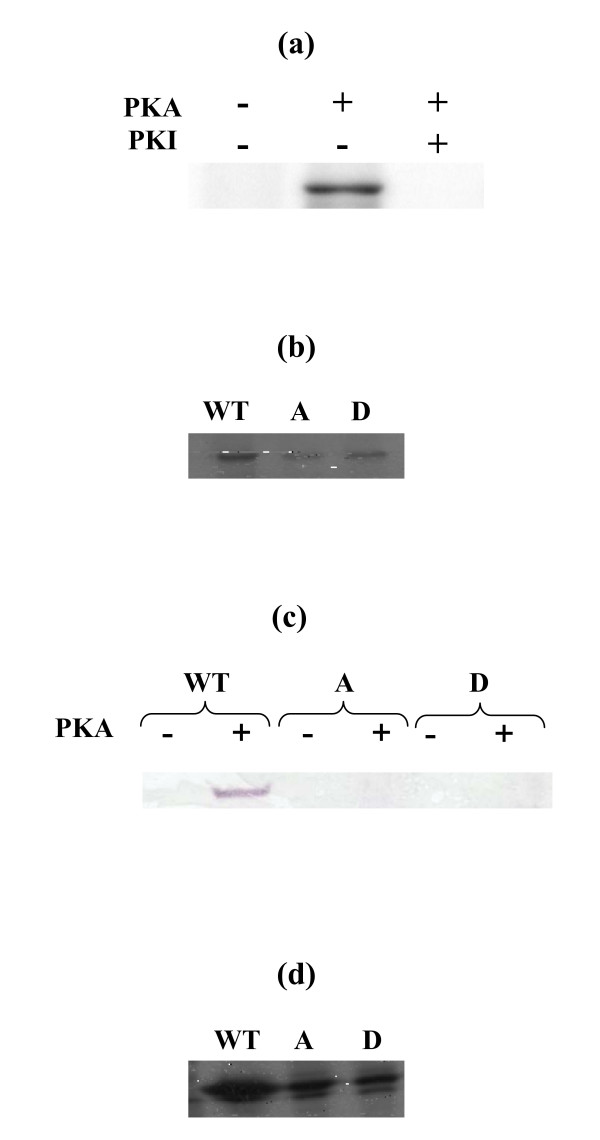
***S. cerevisiae *Sup35p is phosphorylated by PKA *in vitro***. Proteins were incubated with the PKA catalytic subunit and [γ-^32^P]ATP, submitted to SDS-PAGE, and visualized by autoradiography. (a) Phosphorylation of wild-type Sup35p in the presence of PKA or PKI. (b) Phosphorylation of A and D mutant proteins by PKA. (c) Recognition of the different proteins by a specific PKA-phosphorylated substrate antibody. Wild-type, A or D mutant proteins were phosphorylated or not by PKA before western blotting and detection using a secondary antibody linked to the alkaline phosphatase. (d) Phosphorylation of C-terminal proteins by PKA.

His-Tag fusion polypeptides, with either the entire full-length wild-type Sup35p or the A and D mutants, were generated in *E. coli *and purified by Ni^2+^-nitrilotriacetic acid affinity chromatography. The purified proteins were incubated with the PKA catalytic subunit and [γ-^32^P]ATP. Incubation of wild-type Sup35p resulted in the appearance of a ^32^P-labeled species of 78 kDa (the expected apparent molecular mass of Sup35His) (Fig. 2a). Phosphorylation was completely inhibited by PKI, a specific inhibitor of PKA. Incubation of either mutant A or D with PKA and [γ-^32^P]ATP resulted in a reduced phosphorylation. (Fig. 2b). Therefore, the T341 residue of Sup35p is phosphorylated *in vitro *by PKA, and the consensus phosphorylation site of Sup35p is not the only one to be phosphorylated by PKA *in vitro*. This is not surprising as kinases are known to also have non specific activity in *in vitro *conditions.

To verify the phosphorylation state of the T341 residue, *in vitro *phosphorylated wild-type, A and D proteins were submitted to western blot analysis using an antibody specific of substrates phosphorylated by PKA. As expected, only the phosphorylated wild-type protein was detected, the phosphorylated A and D proteins were not, and none of the non-phosphorylated proteins was revealed by the antibody (Fig. 2c).

The expression of full-length Sup35p was relatively poor in *E. coli *cells, while a much greater amount of protein could be recovered from constructs expressing the C-terminal domain alone. Furthermore, the purified full-length proteins were unstable and precipitated rapidly upon storage, probably due to the presence of the aggregating N-terminal domain. Consequently, the same experiments were performed with the C-terminal part of the wild-type, A or D mutant proteins purified as His-Tag fusion polypeptides from *E. coli*. We wondered if the Sup35pcter [C-terminus] alone could be phosphorylated by PKA *in vitro*. The results show that the truncated proteins behaved as did the corresponding full-length proteins. The C-terminal part of Sup35p was phosphorylated by PKA *in vitro*, and the A and D mutants were also phosphorylated but to a much lower extent (Fig. 2d).

To search for a difference in electrophoretic mobility between the *in vitro *phosphorylated and non-phosphorylated protein forms, we performed SDS-polyacrylamide gels in various conditions (see Materials and Methods), but no change in the electrophoretic migration was seen between the protein forms (not illustrated).

### The *in vitro *phosphorylated residue is threonine 341

**Figure 3:**
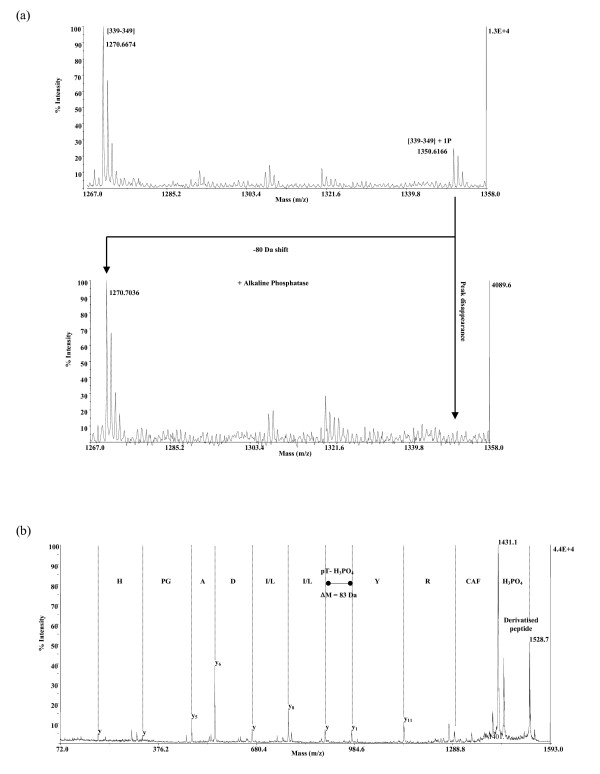
**Mass spectrometry analysis of phosphorylated Sup35p**. (a) On-target dephosphorylation. Detail of the total tryptic digest mass spectra prior and after alkaline phosphatase treatment. The 80 Da shift is the typical signature for a mono-phosphorylated peptide. (b) PSD-MALDI spectrum of the CAF-derivatised peptide [339–349] (HPLC fraction n°22). The neutral loss of H_3_PO_4 _(98 Da) converts pThr into dehydroamino-2-butyric acid produced by β-elimination and the "CAF" gap corresponds to the initial loss of 136 Da from the sulphonic acid group.

To check which residue was phosphorylated by PKA *in vitro*, the phosphorylated full-length His-Tag fusion Sup35p was subjected to mass spectrometry analysis. The MALDI mass spectrum of the unfractioned tryptic digest allowed us to obtain 70% sequence coverage (Fig. 3a). A peptide whose mass did not correspond to the mass of any predicted tryptic peptide was detected, but it was about 80 Da above the theoretical mass of the peptide [339–349]. The possible presence of one phosphate group was fully relevant, as long as the sequence of this tryptic fragment is RYTILDAPGHK, and contains 2 potential phosphorylation sites (Y340 and T341). This was confirmed by performing an on-target dephosphorylation experiment (Fig. 3a). As the signal of the alkaline phosphatase-treated sample was diminished by 80 Da compared to the untreated peptide, [339–349] was indeed a mono-phosphorylated species.

To further investigate the phosphorylation site of the peptide [339–349], we purified the tryptic digest by reversed phase HPLC separation. Each fraction collected was analysed by MALDI mass spectrometry prior to and after on-target dephosphorylation, and [339–349] was recovered in the presence of two other minor peptides (data not shown). Peptide [339–349] of the HPLC fraction was sequenced by PSD-MALDI TOF mass spectrometry. The PSD spectrum of the derivatised peptide [339–349] (m/z 1528.6565) is shown Fig. 3b. The β-elimination occurring (H_3_PO_4 _loss) suggested phosphorylation on the T341 residue rather than on Y340, because such β-elimination is observed for phosphorylated serine or threonine, but not for tyrosine. This was confirmed by the presence of a mass difference between two adjacent peaks corresponding to a dehydroamino-2-butyric acid product (i.e. 83 Da).

### Search for a pool of phosphorylated Sup35p *in vivo*

To determine whether Sup35p is phosphorylated in yeast cells, we labelled with [^32^P]orthophosphate a culture of the *SUP35*-deleted strain that contains a plasmid-encoded *SUP35 *wild-type gene fused to a C-terminal HA-Tag, and immunoprecipitated Sup35p with an HA-specific antibody. Western blot analyses of the immunoprecipitated proteins and the immunoprecipitation supernatants showed that approximately one third of the Sup35 proteins was immunoprecipitated. The same experiments were performed using a strain deleted of PKA, and also in a *SUP35*-deleted strain expressing a plasmid coding for the A mutant protein fused to the HA-Tag and unable to be phosphorylated on T341. Labelling and immunoprecipitation were carried out on exponentially growing cells, as well as on stationary phase cells. In spite of numerous attempts, we did not observe a radioactive pool of Sup35p (data not shown). Since the FS1 strain carries a [PSI+] determinant, it seemed possible that the aggregation of Sup35p could alter protein phosphorylation, or decrease immunoprecipitation efficiency and as a result could reduce the immunoprecipitated sub-fraction of phosphorylated Sup35p. To test this possibility, similar experiments were performed in a *SUP35*-deleted strain carrying only the C-terminal domain of Sup35p, fused to a C-terminal HA-Tag and again, no radioactive pool of Sup35p was detected (data not shown).

### Mutation T341D compromises cell viability, T341A does not

**Figure 4:**
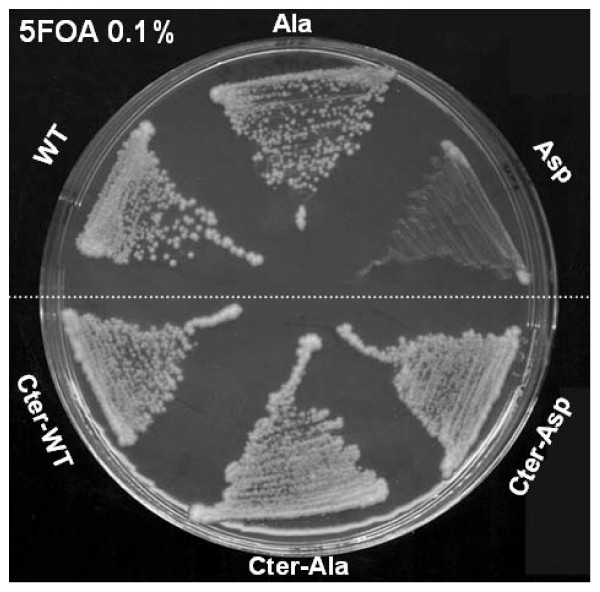
**Full-length T341D compromise viability whereas the C-terminal T341D does not. Both full-length and C-terminal T341A are compatible with viability**. The strain deleted for endogenous *SUP35 *and rescued by an *URA3 *plasmid containing the wild-type *SUP35 *gene was transformed with either pRS313-SUP35, pRS313-SUP35A, pRS313-SUP35D (*HIS3 *plasmids) or with pFL36-SUP35cter, pFL36-SUP35Acter and pFL36-SUP35Dcter (*LEU2 *plasmids). Transformants were streaked onto 5FOA to eliminate the *URA3 *plasmid containing the wild-type copy of *SUP35*.

Complementation assays of a *SUP35 *gene deletion were performed with the mutated alleles. A strain deleted for the endogenous *SUP35 *gene and saved by an *URA3 *plasmid-encoded wild-type *SUP35 *gene was transformed with a *HIS3 *vector carrying either the wild-type *SUP35 *gene, or one of the mutants, or an empty vector. Plasmid shuffling [24] was then carried out by plating the transformants onto medium supplemented with 5-fluoroorotic acid, an uracil analog that allows the formation of colonies only from cells that have lost the wild-type *SUP35 *plasmid with the *URA3 *marker. The ability to form colonies indicates that the Sup35p mutant alone can support viability, while the absence of colonies indicates that the mutant form of Sup35p is unable to support cell viability. The wild-type and the A mutant were able to grow but not the D mutant, suggesting that the T341 residue is subjected to functional constraints and that the introduction of a negative charge in this position alters cell viability. The A mutant behaved like the wild-type strain and did not show thermosensitivity at 37°C (data not shown). A glutamate mutant (T341E) was also constructed to mimic the presence of phosphate groups, and presented the same phenotypes as the D mutant. The essential function of Sup35p is located in the C-terminal domain, thus C-terminal-T341A and C-terminal-T341D mutants were also constructed and used in such a complementation assay as above. Surprisingly, both mutants were able to complement the loss of wild-type Sup35p (Fig. 4), indicating that the N-terminal part of Sup35p might be implicated in the lethality observed in presence of the full-length Sup35p D mutant.

### T341D Sup35p is not degraded *in vivo*

**Figure 5:**
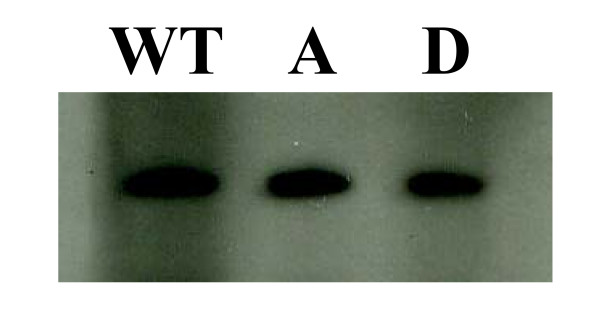
**The T341D Sup35 protein is not degraded *in vivo***. For each strain, the same amount of total proteins was separated by SDS-PAGE and western blot analysis shows the amount of Sup35p in each sample (wild-type (WT), A or D mutants). Detection was made using a primary antibody specific of the M domain of Sup35p, and a secondary antibody fused to the horseradish peroxydase.

To check if the lethality of the D mutant was due to the degradation of the protein, western blot analyses were carried out to monitor the steady-state levels of the mutant forms of Sup35p. Protein extracts were obtained from *SUP35*-deleted strains carrying either one of the mutated full-length alleles on a centromeric *HIS3 *plasmid (saved by the plasmid-encoded C-terminal part of the *SUP35 *wild-type gene). To permit valid comparisons, the amounts of total protein extract loaded per well were the same. The results indicate that each mutant form of Sup35p was present at a steady-state level similar to that of the wild-type Sup35p (Fig. 5). Thus the incapacity of the full-length D mutant to grow does not result from Sup35p degradation.

### The Sup35pcter T341A and T341D mutations decrease the efficiency of translation termination *in vivo*

Mutations in the gene encoding eRF3 have frequently been reported to exhibit an omnipotent suppressor phenotype, where readthrough is thought to increase at all stop codons [16,25]. We examined how mutation of T341 affected the efficiency of translation termination at each of the three stop codons by using a dual reporter system [26]. The dual reporter plasmids contain an upstream *lacZ *gene and a downstream firefly luciferase gene separated by different in-frame stop codons in the Tobacco Mosaic Virus readthrough context. These constructs made it possible to monitor the translation readthrough of different termination signals in different genetic contexts by measuring firefly luciferase activity, while the β-galactosidase activity served as an internal normalization control.

**Figure 6:**
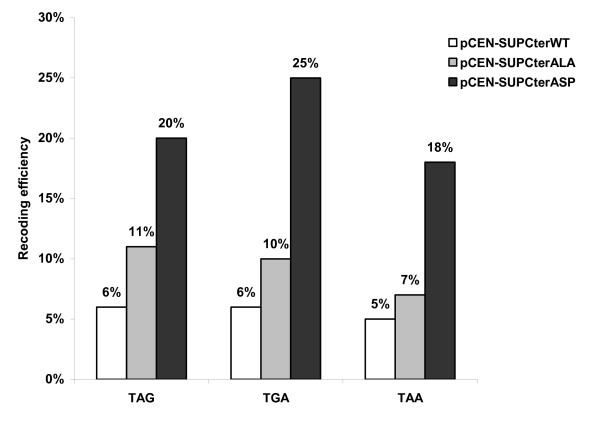
***In vivo *translation termination efficiency of T341A Sup35pcter and T341D Sup35pcter**. Percent readthrough of the three different stop codons (TAG, TGA and TAA) carried on a dual reporter system was estimated in strains expressing the C-terminal Sup35p either wild-type, A mutant or D mutant, as sole source of Sup35 protein.

The *SUP35*-deleted strain saved by either a plasmid-encoded C-terminal wild-type, C-terminal A mutant or C-terminal D mutant gene was transformed with the dual reporter constructs, readthrough activities were measured and statistical analyses using the Mann-Whitney non parametric test were performed [27]. Yeast strains expressing the C-terminal A or D Sup35p as the sole source of Sup35 protein exhibited a significantly increased readthrough level compared to cells expressing wild-type Sup35p (Fig. 6). The readthrough efficiency of the A mutant was 1.4 to 1.8 fold higher than the wild-type (P value TAG = 0.00333, TGA = 0.00177, TAA = 0.00177). The D mutant showed a stronger increase in readthrough with increase factors ranging from 3.4 for the TAG codon (P value = 0.00214) and 3.6 for the TAA codon (P value = 0.00044), to 4.2 (P value = 0.00044) for the TGA codon compared to the wild-type C-terminal Sup35p.

### GTPase activity of the A and D mutants

The Sup45p- and ribosome-dependent GTPase activity of the A and D mutant Sup35pcter was checked *in vitro *by accumulation of [^32^P]P_i _using the modified charcoal precipitation assay as described [7]. The D mutant was practically inactive in the GTPase assay whereas the A mutant exhibited residual GTPase activity (6% compared to the wild-type Sup35pcter) (Table 1).

**Table 1:** *In vitro *Sup45p- and ribosome-dependent GTPase activity of A and D mutant Sup35pcter.

**Components of the incubation mixture**	**^32^P[P_i_] released (pmol)**	**Activity in % compared to the WT**
Sup45p	0	-
Sup35pcter wild-type	0	-
Sup35pcter wild-type + Sup45p + ribosomes	2.2	**100%**
A mutant Sup35pcter + Sup45p + ribosomes	0.14	**6%**
D mutant Sup35pcter + Sup45p + ribosomes	0.02	**1%**

### Interaction of A and D Sup35 proteins with Sup45p (eRF1)

**Figure 7:**
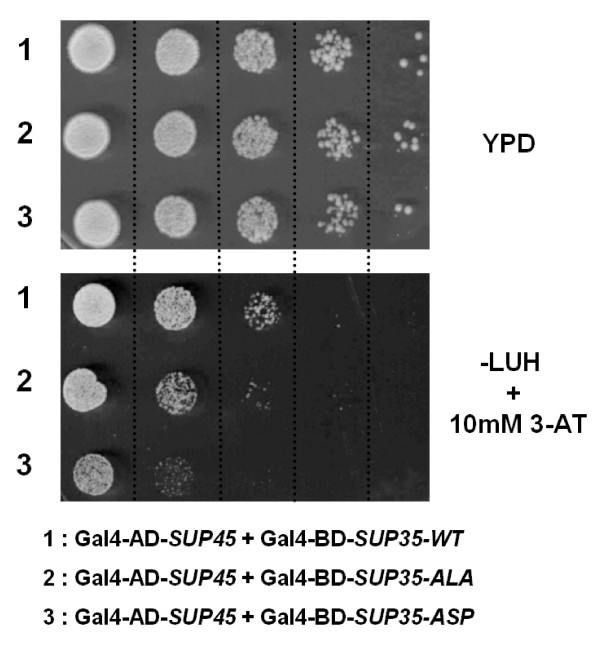
**Interaction between Sup45p and wild-type, A or D mutant Sup35 proteins in a two-hybrid system**. The entire *SUP35 *gene was fused to the binding domain of Gal4 (Gal4-BD), and the *SUP45 *gene to the activating domain of Gal4 (Gal4-AD). Serial dilutions of diploids were spotted on YPD rich medium and on a medium lacking leucine, uracile and histidine with 10 mM 3-aminotriazol to reveal interactions.

Translation termination is triggered by a complex consisting of Sup45p and Sup35p [9]. We analyzed the capacity of the A and D mutant proteins to interact with the Sup45p partner by a two-hydrid approach. Interactions were visualized by growth on a medium lacking histidine with 10 mM 3-aminotriazol added. The two mutant Sup35 proteins could still interact with Sup45p, but with various efficiencies. The results indicated strong interaction between the wild-type proteins, decreased interaction between Sup45p and the Sup35p A mutant, and the weakest between Sup45p and the Sup35p D mutant (Fig. 7). The frequency of diploid cells interacting with each others was estimated for these three diploids. Three independent cultures were prepared in minimal medium lacking uracile and leucine for each diploid, and plated onto either a minimal medium lacking histidine, uracile and leucine to count the cells presenting a Sup35p and Sup45p interaction, or a minimal medium lacking uracile and leucine to count diploid cells. The results indicated an interaction in 64% of the cells between the wild-type proteins, in 22% of the cells between the Sup35p A mutant and Sup45p, and in 15% of the cells between the Sup35p D mutant and Sup45p.

A similar picture of various interaction efficiencies of the A and D mutant Sup35pcter with Sup45p was also observed in another *in vitro *assay. It was shown that Sup35p can stimulate the RF activity of Sup45p in an *in vitro *system at low stop codon concentration while Sup45p alone at the same concentration of stop codon is inactive [8,28]. The stimulation of Sup45p activity in the presence of Sup35p might be caused by mutual interaction of these proteins. The RF stimulating activity of Sup35pcter towards Sup45p was measured as described [8]. The stimulating RF activities of the A and D mutant Sup35pcter towards Sup45p were 60 and 35%, respectively compared to the wild-type Sup35pcter activity (not shown).

## Discussion

The C-terminal domain of Sup35p, implicated in translation termination, contains a consensus site for phosphorylation by PKA. Alignment of eRF3 sequences showed that this site is highly conserved. Kallmeyer *et al*. [29] already addressed the absence of phosphorylation of Sup35p *in vivo*, however Le Roy *et al*. [30] demonstrated differential phosphorylation states of the human eRF3, which is highly homologous to the yeast Sup35p. In addition, the serine/threonine protein kinase Sch9, whose activity functionally overlaps those of the PKA and TOR kinases, was recently identified in a proteome-wide screen for protein-protein interactions as forming a complex with, and presumably, phosphorylating Sup35p [31].

Phosphorylation being sometimes very labile, the modified isoforms might be difficult to detect. This led us to run the experiment in conditions different from Kallmeyer *et al*. We demonstrated that threonine 341 of the consensus site was indeed phosphorylated *in vitro *by PKA. However, in the *in vivo *conditions tested, no pool of phosphorylated Sup35p was observed. Since Budovskaya *et al*. [23] demonstrated that the presence of a highly conserved PKA consensus site was a very strong predictor of physiological PKA phosphorylation, we think it cannot be excluded that phosphorylation could occur, but in particular conditions we did not explore yet. However, the simplest interpretation is that Sup35 protein is actually not phosphorylated *in vivo*. Indeed, *in vitro *phosphorylation assays frequently identify non-physiological substrates for various kinases.

Although no phosphorylation was detected in position 341, mutant forms of Sup35p, mimicking either a non-phosphorylated (A mutant), or a constitutively phosphorylated (D mutant) protein were constructed, anticipating that if phosphorylation actually occurs in particular physiological conditions, the residue involved would have an important role in the function of the protein. These mutations were incorporated either in the full-length protein or in the essential C-terminal domain. An almost two fold increase in the level of readthrough was observed with the C-terminal form of the T341A mutant and a four fold increase was observed with the C-terminal form of the T341D mutant, compared to the wild-type. Consistent with these results, both mutant forms showed decreased interaction with Sup45p, indicating that the termination complex is less actively formed. The highest readthrough levels obtained in the A and D mutants were observed with the TGA stop codon. This observation is consistent with the hypothesis of Salas-Marco and Bedwell [10] that Sup35p may help Sup45p discriminate between UGG and certain stop codons.

When we checked for phenotypes, we found that strains expressing the C-terminal form of either mutant are viable, while the T341D mutation in the full-length protein context was not able to support viability. Thus functional interaction between the N- and C-terminal parts of Sup35p is responsible for the lethality of the full-length D mutant. The presence of the N-terminal part of Sup35p was previously shown to decrease translation termination efficiency in a [psi-] background [32]. Similarly, in a recent work Volkov *et al *[33] found that the N terminus acts as a negative factor, increasing nonsense suppression efficiency in *sup35 *mutants containing amino acid substitutions in the C-terminal domain. Therefore, one reason for the lethal effect of the T341D mutation in the full-length protein could be a too important rate of nonsense readthrough, which could be incompatible with cell viability. Strikingly, a Y340H substitution (the residue preceeding T341) directed higher stop codon readthrough efficiencies in a full-length compared to a C-terminal context, while not affecting the PKA consensus site. This is consistent with the results reported here for the T341D mutant. In the later case the consequence is even more drastic since the presence of both domains leads to lethality. Volkov *et al*. [33] concluded that the N-terminal extension of Sup35p might decrease the GTPase activity in strains that contain amino acid substitutions in the C-terminal domain. In our case, the GTPase activity of the C-terminal D mutant is already extremely low, but if it is sufficient for cell growth, adding the N-terminal domain could reduce it to a non viable level.

The T341 residue is located in the GTPase fold of Sup35p, between the switch I and G2 switch II regions. The A and D mutations most likely affect the Sup45p and ribosome-dependent GTPase function of the resulting proteins, since both of them display reduced GTPase activity *in vitro*. The A mutation exhibits a strong reduction of GTPase activity, and to date, the GTPase activity of Sup35p was always shown to be essential for cell viability [10]. In the C-terminal D mutant, the GTPase activity is 100 times lower than in the wild-type but it is still compatible with growth. Cell viability of both the full-length and the C-terminal A mutant could be explained by the residual GTPase activity (about 15 times less compared to the wild-type Sup35p), since the concentration of Sup35p in yeast is 6 times higher than the concentration of Sup45p (1.31 10^4 ^protein molecules per cell for Sup45p and 7.89 10^4 ^for Sup35p [34]). These results lead to two interesting conclusions: 1) even with a 100 times lower GTPase activity, this C-terminal D mutant is still active in termination and supports viability; 2) there must be a negative feedback link between this mutation in the C-terminal domain and the N-M domains of Sup35p.

Salas-Marco and Bedwell [10] examined how mutations in the GTPase domain of Sup35p influence translation termination, and suggested that the GTPase activity of Sup35p is required to couple recognition of translation termination signals by Sup45p to efficient polypeptide chain release. One of the mutants these authors described (H348L), which is unable to support viability and has the largest reduction in GTPase activity, could correspond to our D mutant, but none of the other mutants was comparable to the A mutant, with a growth similar to the wild-type and a low GTPase activity, nor to the C-terminal D mutant with its extremely low GTPase activity. Recent findings shed light on the role of Sup35p in translation termination [9]: based on *in vitro *evidences a model is proposed where the GTPase activity is needed for the correct positioning of the Sup45p GGQ in the PTC. This might lead to the release of Sup35p-GDP from the ribosome or modify the interaction between Sup35p-GDP and Sup45p. Sup45p would then trigger hydrolysis of the peptidyl-tRNA. Our *in vitro *results are consistent with this possibility since the stimulating RF activities of the A and D mutant Sup35pcter towards Sup45p were clearly diminished.

Differences in protein activities between the mutants studied here most likely depend on the structure of Sup35p. Only a small effect is observed on the interaction of the mutated proteins with its Sup45p partner, but it is not an "all or nothing" response, and is probably due to a change in protein conformation. According to the crystal structure available for eRF3 of *Schizosaccharomyces pombe *[17], the S319 residue, corresponding to *S. cerevisiae *T341, is not directly involved in GTP binding but localizes at the surface of the protein. Due to their high flexibility, large portions of the switch I and II regions of the GTPase domain were not defined by electron density and hence are absent from the crystal structure (AA 280–307 and 325–331 are missing). It is therefore difficult to precisely predict the impact of the mutations studied on the protein structure. This region was speculated to become ordered when the Sup45p/Sup35p-GTP complex associates with the ribosome, thereby stimulating the GTPase activity of Sup35p in the presence of Mg^2+^. In G proteins, it is well documented that these switch regions adopt different conformations depending on the nucleotide bound (GDP or GTP) and on the coordinate Mg^2+^, which is an essential cofactor for GTP hydrolysis [35]. The D mutation might influence both regions, resulting in a dramatic conformational change and loss of GTP hydrolysis activity.

It has also been suggested that Sup35p might be implicated in other pathways different than translation processes [32,36,37]. Recent data focused on the relation between Sup35p and the cytoskeleton since it interacts through its N-terminal domain with various proteins of the actin cortical cytoskeleton. The T341D mutation might thus lead to an altered protein unable to perform these other functions. Since the interaction between Sup35p and the cytoskeleton is not known to involve its GTPase activity, it might be this pathway that is involved in the essentiality of Sup35p. This would explain why the D mutant, almost completely devoid of GTPase activity and highly deficient in termination, is nevertheless viable.

## Conclusion

In the present study, we characterized T341 as a new critical residue for peptide release factor Sup35p activity. We demonstrate that this residue is involved in the activity of Sup35p in translation termination and functionally interacts with the N-terminal part of the molecule. That an extremely low GTPase activity of Sup35p is compatible with normal growth raises interesting questions about the absolute requirement of this release factor for cell viability. Indeed the indispensability of the protein was so far related to its GTPase activity, and our work demonstrates either that extremely low GTPase activity is sufficient for cell growth, or that the protein is implicated in one or several other essential pathways not requiring the GTPase activity.

## Methods

### Strains, growth conditions and methods

The *S. cerevisiae *strains used here were FS1 (MAT α ade2-592 (frameshift) *lys2Δ201 leu2-3, 112 his3Δ-200 ura3-52*) which is derived from Y349 [38], and pJ69-4A for the two-hybrid analysis (MAT *a *and *α trp1-901 leu2-3, 112 ura3-52 his3Δ-200 gal4Δ gal80Δ GAL2-ADE2 LYS2::GAL1-HIS3 met2::GAL7-lacZ*) (described in [39]). Standard rich (YPD) or synthetic (SC) culture medium was prepared as described [40]. The *E. coli *strain DH5*α *(*supE44 Δlac U169 (Φ80 lacZΔM15) hsdR17 recA1 endA1 gyrA96 thi-1 relA1*) was used for cloning experiments, and was cultivated in LB medium [41]. Appropriate amounts of antibiotics, amino acids and bases were added when necessary. Yeast transformation was performed by the lithium acetate method as described previously [42]. *E. coli *cells were transformed as indicated [43]. For plasmid shuffling [44], a selective medium containing 0,25% 5-fluoroorotic acid (5-FOA, Fermentas) was used.

### Gene disruption

The FS1 *sup35*Δ strain was generated by a one-step gene replacement method. The *SUP35 *gene was disrupted by the removal of the entire open reading frame and the insertion of the *ADE2 *gene. Y349 genomic DNA was used as template to generate the PCR fragment carrying the *ADE2 *gene plus the promoter with the sense (5'-TTCTTCATCGACTTGCTCGGAATAACATCTATATCTGCCCACTAGCAACAAACACC AACATAACACTGACATC) and reverse (5'-CTTTATGATCGGTATTATTGTGTTTGCATTTACTTATGTTTGCAAGAAATGGACAC CTGTAAGCGTTGATTTC) primers, which contain extensions corresponding to the flanking upstream and downstream sequences of the *SUP35 *open reading frame, respectively. The upstream (sense 5'-CGAGAAGATATCCATCATATTACC and reverse 5'-TGTTGCTAGTGGGCAGATATAG primers) and downstream (sense 5'-ATTTCTTGCAAACATAAGTAAATG and reverse 5'-GCTTCTGAAGATAGATGAGCGG primers) regions of the *SUP35 *gene were amplified by PCR from FS1 genomic DNA. The three PCR fragments were then joined by running a PCR reaction with each of them in equimolar amounts, resulting in the final disruption fragment. The yeast FS1 strain was transformed with the disruption fragment and the centromeric *URA3 *plasmid pRS316-SUP35. Ura^+ ^Ade^+ ^transformants (white colonies) were tested for the ability to evict the *URA3 *plasmid on 5-FOA plates. Clones which were unable to grow on 5-FOA medium had integrated the *ADE2 *gene on the chromosome, and not on the pRS316-SUP35 plasmid. The correct genomic integration event was then verified by PCR on the genomic DNA.

### Plasmids

Centromere- and 2μ DNA-based plasmids were used to express wild-type and mutant forms of *SUP35 *in yeast. The centromeric *URA3 *plasmid pRS316-SUP35 was kindly provided by Dr. M.D. Ter-Avanesyan (Institute of Experimental Cardiology, Moscow). The pRS316-SUP35A, pRS316-SUP35D, and pRS316-SUP35E constructs with the mutant T341A (ACC to GCC change using the sense 5'-AAAAAGGCGTTATGCCATATTGGATGCTCC and antisense 5'-GGAGCATCCAATATGGCATAACGCCTTTTT primers), T341D (ACC to GAC change) and T341E (ACC to GAA change) alleles (same primers except for the mutated codon), respectively, were obtained by site-directed mutagenesis of the PKA consensus site threonine 341, using the QuikChange site-directed mutagenesis kit (Stratagene). The centromeric *HIS3 *plasmids were constructed by insertion of the *XbaI-XhoI *fragments, containing the wild-type or mutant *SUP35 *genes under the control of the *SUP35 *promoter into the same sites of pRS313. The 2μ DNA-based *TRP1 *plasmids were obtained by insertion of the *SacI-XhoI *fragments, carrying the *SUP35 *promoter and wild-type or mutated *SUP35 *genes, into the *SacI-SalI *sites of pFL45L. The centromeric *LEU2 *plasmid pFL36-SUP35cter expressing the C-terminal part of wild-type Sup35p (amino acids 254 to 685) under the *SUP35 *promoter, was kindly provided by Dr. O. Namy (Institut de Génétique et Microbiologie, Orsay). The *StuI-NcoI *fragment from pFL36-SUP35cter, carrying the T341, was replaced by the same fragment obtained from pRS313-SUP35A or pRS313-SUP35D, resulting in pFL36-SUP35Acter and pFL36-SUP35Dcter, to express the C-terminal domain of the A or D mutants, respectively. The *EcoR1-BamH1 *fragments from the three pFL36 vectors were inserted into the *EcoR1-BamH1 *cleaved pHS8 plasmid (2μ-based DNA, *HIS3 *marker) to overexpress the wild-type, A or D, C-terminal part of eRF3.

To express the entire wild-type, A and D Sup35p HA proteins in yeast, the corresponding open reading frames (without the stop codon) were amplified using the direct (5'-CACTAGAATTCATGTCGGATTCAAACCAAGGC) and reverse (5'-CAAGAGGATCCTCGGCAATTTTAACAATTTTACC) primers containing respectively the *EcoRI *and *BamH1 *sites (underlined), and cloned into the *EcoRI-BamH1 *sites of the pYX212 vector (*URA3*, 2μ-based plasmid – R&D Systems, Mineapolis USA) under the control of the constitutive TPI promoter. Constructs for the expression in yeast of the C-terminus SUP35 HA proteins were generated by inserting the PCR-amplified 3'-fragments of the *SUP35HA *gene from pYX212 with the direct (5'-CACGCGCTATTGGCCAAGACCCAAGG) and reverse (5'-GAACAGCTCTAGAGATATCATGCGTAGTCAGGCTC) primers containing respectively the *MscI *and *XbaI *sites (underlined), and cloned into the pFL36-SUP35cter vector digested by *MscI-XbaI*.

pET-SUP35 for the expression of His_6_-tagged Sup35p in *E. coli *was produced by inserting a PCR fragment amplified from pRS316-SUP35 with the direct (5'-AGATATACATATGTCGGATTCAAACCAAGGC) and reverse (5'-GCCGGATCCTTACTCGGCAATTTTAACAATTTTACC) primers containing extensions with *NdeI *and *BamH1 *sites (underlined), respectively, into the *NdeI-BamH1 *cleaved pET-28a vector (Novagen). The resulting construct contains the 685 codons of *SUP35 *fused in frame to the 6 His codons at the N terminus. The pET-SUP35A and pET-SUP35D plasmids for the expression of the entire A and D eRF3 mutants in *E. coli *were obtained by replacing in pET-SUP35 the *MscI *fragment containing the mutation position by the same fragment obtained by digestion of pRS316-SUP35A and pRS316-SUP35D, respectively. The pET-SUP35cter plasmid (pYSC2) was kindly provided by Dr. R.H. Buckingham (Institut de Biologie Physico-Chimique, Paris). The pET-SUP35Acter and pET-SUP35Dcter, expressing in *E. coli *the N-terminal His_6_-tagged eRF3 A or D C-terminus, respectively, were constructed by replacing in pET-SUP35cter the *NcoI-StuI *fragment by the corresponding fragments from pRS313-SUP35A or pRS313-SUP35D.

The plasmid for expression of Sup45p in *E. coli *was created on the basis pET30a and kindly provided by V.N. Urakov (Institute of Experimental Cardiology, Moscow).

The 2μ-based pGAD-C3 (*LEU2 *marker) and pGBDU-C3 (*URA3 *marker) vectors [39] were used to generate plasmids for the two-hybrid analysis. The pET-SUP35 vector was digested by *NdeI*, then Klenow-filled, and digested by *BamH1*. The resulting fragment carrying the entire *SUP35 *open reading frame was cloned into the *SmaI-BamH1 *sites of the pGAD-C3 and pGBDU-C3 plasmids. The Gal4 activating and binding domain fusions with the A and D Sup35 mutants were obtained by replacing the wild-type *NcoI-StuI *fragments by the mutated fragments digested from pRS313-SUP35A and pRS313-SUP35D. For *SUP45*, a PCR fragment amplified from FS1 genomic DNA with the direct (5'-CCATCGATGGATAACGAGGTTGAAAAAAATATTG) and reverse (5'-TTTTCTGCAGTTAAATGAAATCATAGTCGGATCC) primers containing extensions with *ClaI *and *PstI *sites (underlined), respectively, was cloned in frame into the *ClaI-PstI *cleaved pGAD-C3 and pGBDU-C3 vectors. The resulting plasmids overexpress Sup45p fused to either the Gal4 activating or Gal4 binding domains.

### Purification of eRF3 and eRF1 proteins

Sup35 and Sup35cter proteins were purified as follows. Expression was performed at 37°C using the transformed *E. coli *Rosetta strains (Novagen) and LB medium supplemented with 17 μg/ml chloramphenicol and 50 μg/ml kanamycin. When the cell culture reached an OD_600 nm _of 0.5, protein expression was induced with 0.5 mM IPTG (Sigma) and the cells were grown for a further 3 h. The cells were harvested by centrifugation and stored at -20°C. The cell pellet was resuspended in 40 ml of 20 mM Tris-HCl pH 7.5, 150 mM NaCl, 5 mM β-mercaptoethanol, and protease inhibitors. Cell lysis was completed by sonication. The His-tagged protein was purified on an Ni-NTA column (Qiagen Inc.), eluted with imidazole, and loaded onto a SuperdexTM200 column (Amersham Pharmacia Biotech) equilibrated with 20 mM Tris-HCl pH 7.5, 150 mM NaCl, and 10 mM β-mercaptoethanol. The homogeneity of the proteins was checked by SDS-PAGE.

The Sup45p with 6 His-residues on the N terminus was expressed in *E. coli *and purified on Ni-NTA resin (Qiagen).

### *In vitro *phosphorylation

Aliquots (2 μg) of purified N-terminally His_6_-tagged Sup35 and Sup35cter proteins were added to a PKA reaction buffer containing 25 mM Tris pH 7.5, 10 mM MgCl_2_, and 2 μCi of [γ-^32^P]ATP (3000 Ci/mmol), and incubated with 2.5 U of the PKA catalytic subunit (Sigma) at 30°C for 30 min in a total volume of 40 μl. To test for the specificity of the reaction for PKA, 30 μg of PKA inhibitor (PKI) (Sigma) were added to the reaction buffer to specifically inhibit the activity of PKA. For mass spectrometry analyses, Sup35p (100 μg) was phosphorylated by 75 U of PKA with 750 μM unlabelled ATP in 1.2 ml. Phosphorylated proteins were then TCA precipitated and loaded onto 10% SDS-polyacrylamide gels, which were then autoradiographed. For mass spectrometry analyses, the gels were stained with Coomassie Blue, and the bands of interest were excised for further analysis.

To investigate differences in electrophoretic mobility between the *in vitro *phosphorylated and non-phosphorylated protein forms, samples were loaded onto various SDS-polyacrylamide gels and revealed by Coomassie staining: 4–12% gradient polyacrylamide gel, or 10% acrylamide/0.1% bis-acrylamide gel, or 10% ProSieve 50 gel, which is a modified acrylamide formulation developed for high performance protein gel electrophoresis (TEBU).

### Mass spectrometry analysis

#### In-situ proteolysis

Protein bands were excised, sliced into 1 mm cubes, thoroughly washed with water and dehydrated in acetonitrile for 10 min at 37°C. They were destained by several incubations in 100 mM ammonium bicarbonate pH 8.5 containing 50% acetonitrile, dehydrated again in acetonitrile and vacuum dried. After reduction (10 mM DTT for 35 min at 56°C) and alkylation (55 mM iodoacetamide for 30 min at room temperature), each cube was reswollen in 20 μl 50 mM ammonium bicarbonate pH 8.5 containing 10% acetonitrile and 500 ng of trypsin (unmodified, sequencing grade, Roche Diagnostics), and left on ice for 45 min before proteolysis (overnight at 37 °C). Supernatants were removed and peptides extracted sequentially with 1% trifluoroacetic acid (TFA) and followed 1% TFA in 60% acetonitrile. The resulting supernatants were combined and dried.

#### Peptide purification

Tryptic peptides resuspended in 0.1% TFA were loaded onto a 250 × 2,1 mm Vydac C18 reversed phase column (300 Å pore size) and eluted with a 90 min linear gradient of 0–70% acetonitrile in 0.1% TFA. The flow rate was 0.3 ml/min. Fractions were collected manually based on the absorbance profile at 215 nm.

#### MALDI-TOF mass spectrometry analysis

An aliquot of unfractionated tryptic digest was resuspended in 10 μl 50% acetonitrile and 0.3% TFA, and 1 μl was mixed in a 1:1 ratio with a saturated solution of a-cyano-4-hydroxycinnamic acid in 0.3% TFA/50% acetonitrile. An aliquot of each HPLC fraction was concentrated 20-fold under vacuum before 1 μl of the concentrate was mixed with 1 μl of the matrix saturated solution. The different mixes were deposited on a standard stainless probe and allowed to air dry. MALDI analysis was performed on a Voyager-DE STR equipped with a 337 nm laser source (Applied Biosystems, USA). Spectra were recorded in positive-ion reflectron mode, with a 20 kV acceleration voltage in the ion source. External calibration was performed with a mixture of 6 reference peptides covering the m/z 900–3700 Da range, allowing a mass accuracy of 10 to 30 ppm.

#### Phosphopeptide and phosphorylation site identification

To ensure a reliable identification of phosphorylated peptide(s), dephosphorylation experiments were performed for unfractionated tryptic digests and HPLC fractions on the MALDI target using alkaline phosphatase (1 unit/μl; Roche) prior to analysis as described [45]. To characterize the phosphorylation site in peptide [339–349], a Post-source-decay MALDI-TOF (PSD) experiment was performed. As tryptic peptides are difficult to fragment in PSD-MALDI-TOF due to their proton sequestring C terminus, the Chemically Assisted Fragmentation (CAF) technology was used. Based on the introduction of a negatively charged sulphonic acid group to the N terminus of peptides after lysine residue protection by their conversion to homoarginine, this technology significantly improves the sensitivity of PSD-MALDI by increasing the fragmentation efficiency. Furthermore, the clarity of the spectrum is improved as only the y-ion series is observed. Indeed, only the y-ions (C-terminal fragments) retain a net positive charge allowing them to be detected, while the b-ions (N-terminal fragments) are neutral and cannot be detected. Simplified mass data were obtained using the Ettan CAF MALDI sequencing kit as described by the manufacturer (GE Healthcare Amersham Biosciences).

### Western blots

Protein samples were separated on SDS-polyacrylamide gels and electrophoretically transferred to nitrocellulose membranes [46]. Western blots were probed with either affinity purified polyclonal rabbit antibodies against the peptide 137–151 of Sup35p (a kind gift from Dr. S. Lindquist), or a rabbit monoclonal antibody against phospho-PKA substrates (detecting R-R-X-S*/T* motifs, Cell Signaling). The bound antibodies were detected using the Amersham ECL system.

### *In vivo *labelling and immunoprecipitation

Yeast cells were grown in a low-phosphate YPD medium (LP-YPD Broth, BIO 101 Systems) to the appropriate OD_600 _(1.5 for the exponential phase, and 6 for the stationary phase). Cells in 1 ml growth medium, harvested from 4 ml for the exponential and 1 ml for the stationary phases were labeled by adding 250 μCi of [^32^P] orthophosphate (Amersham) for 1 h at 30°C. The cells were lysed by incubation for 10 min on ice with 100 μl of 1.85 M NaOH plus 1% β-mercaptoethanol. Proteins were then precipitated by adding 100 μl of 50% TCA. The precipitate was centrifuged at 15,000 g for 5 min, washed with 1 M Tris base, resuspended in 50 μl of SDS-PAGE loading buffer without β-mercaptoethanol [47], and dissociated by heating for 10 min at 95°C. TNET buffer (0.6 ml; TNET = 50 mM Tris-HCl pH 7.4, 150 mM NaCl, 5 mM EDTA, 1% Triton X-100, plus 1 mM phenylmethylsulphonyl fluoride (PMSF) and protease inhibitors) was added, and the insoluble material was removed by centrifugation for 30 min at 12,000 g. The supernatant was incubated overnight at 4°C with 50 μl rat monoclonal anti-HA antibody (clone 3F10, Roche Diagnostics), plus 100 μl of 50% protein G-Agarose beads (Roche Diagnostics). Supernatants were 10-fold concentrated using Vivaspin 500 ultrafiltration spin columns (10,000 MWCO PES, Sartorius AG), and aliquots were loaded on 10% SDS-PAGE gels. Immunoprecipitates were washed twice with TNET buffer, twice with 100 mM Tris-HCl pH 7.5, 300 mM NaCl, and once with 25 mM Tris-HCl pH 7.5. The proteins were eluted by incubation for 5 min at 95°C in 100 μl of 2-fold concentrated SDS-PAGE loading buffer. Samples were resolved by 10% SDS-polyacrylamide gels and autoradiographed.

### Quantification of readthrough efficiency

Yeast strains were transformed with pAC99 reporter family plasmids. In each case, at least five independent transformants, cultivated in the same conditions, were assayed. Cells were broken using acid-washed glass beads, as described [26]. Luciferase and β-galactosidase activities were assayed in the same crude extract. Readthrough efficiency is estimated by the ratio of luciferase activity to β-galactosidase activity. To establish the relative activities of β-galactosidase and luciferase when expressed in equimolar amounts, the ratio of luciferase activity to β-galactosidase from an in-frame control plasmid was taken as a reference. Readthrough frequency, expressed as percentage, was calculated by dividing the luciferase/β-galactosidase ratio obtained from each test construct by the same ratio obtained with the in-frame control construct [24]. This allowed us to determine the percent readthrough while controlling differences in mRNA abundance or efficiency of translation initiation.

### Two-hybrid analysis

The GAL4-based two-hybrid system [39] was used. pGAD plasmids (empty pGAD, pGAD-SUP35, pGAD-SUP35A, pGAD-SUP35D and pGAD-SUP45) were transformed into the MATα pJ69-4A strain, and pGBDU plasmids (empty pGBDU, pGBDU-SUP35, pGBDU-SUP35A, pGBDU-SUP35D and pGBDU-SUP45) into the MATa pJ69-4A strain. These transformants were mated in different pairwise combinations overnight at 30°C on YPD plates, and diploids selected on uracil and leucine omission medium. The two-hybrid interactions were assessed by the ability to transactivate *HIS3 *or *ADE2 *reporter genes (to confer growth either in the absence of adenine, or in the absence of histidine and in presence of different concentrations of 3-amino-1,2,4-triazole (Sigma)). Interactions were visualized on histidine, uracil and leucine omission medium containing either 5 mM, 10 mM, or 25 mM 3-amino-1,2,4-triazole, and also on the more selective adenine, uracil and leucine omission medium. Similar interactions were detected on each medium when Sup35 was fused to the Gal4 activating domain (pGAD vectors) and Sup45 to the binding domain (pGBDU vectors). Lighter interactions were seen in the opposite combinations (Sup35 into pGBDU vectors and Sup45 into pGAD vectors) on the histidine omission medium, and no interaction at all on the adenine omission medium. As negative control, diploids bearing the empty vector and fusion constructs were used, and the *HIS3 *or *ADE2 *reporter genes were not activated. Quantitative β-galactosidase assays were undertaken on lysates prepared from each diploid strain in order to estimate the interaction strength, but no coherent results were obtained because of the leakiness of the promoter controlling the expression of the *lacZ *gene (same level of β-galactosidase activity in the diploid carrying at least one empty vector).

### Assay for GTPase activity

The incubation mixture (12.5 μl) contained 2 μM [γ-^32^P] GTP (sp. activity 4000–5000 cpm/pmole), 20 mM Tris-HCl, pH 7.5, 30 mM NH_4_Cl, 15 mM MgCl_2_, 0.2 μM 80S rabbit ribosomes, 0.32 μM Sup45p and 0.4 μM Sup35pCter. The reaction was performed at 25°C for 30 min, stopped by adding 750 μl of 5% charcoal in 50 mM NaH_2_PO_4 _on ice. The mixture was vortexed and centrifuged at 10,000 rpm for 10 min at 4°C. The [^32^P]P_i _released into 500 μl of supernatant was quantitated by liquid scintillation counting. [^32^P]P_i _release in the absence of one out of the three protein components of the incubation mixture was measured and the average value (8–10%) was subtracted for all samples.

### The RF stimulating activity of Sup35pcter towards Sup45p in an *in vitro *termination assay

The RF stimulating activity of Sup35pcter towards Sup45p was measured as described [8]. Stop-codon-dependent hydrolysis of f[^35^S]Met-tRNA^fMet ^associated with the AUG-80S rabbit ribosome complex took place at 5 μM stop codon (UAAA) that corresponded to ~10% of the saturation level needed for complete fMet release with Sup45p but without Sup35pcter. The RF activity was calculated as the amount of f[^35^S]Met released in the presence of stop codon; the value of f[^35^S]Met released in the absence of stop codon was subtracted from all values.

## Authors' contributions

CF, BC and IH performed the molecular characterization of the mutants and the phosphorylation experiments. SL carried out the GTPase and termination assays. SG and PL performed the mass spectrometry analysis. JPR coordinated the project. All authors read and approved the final manuscript.
